# National Trends in Total Hip Arthroplasty Bearing Surface Usage in Extremely Young Patients Between 2006 and 2016

**DOI:** 10.1016/j.artd.2021.05.017

**Published:** 2021-07-09

**Authors:** Christopher M. Hart, Clark Chen, Peter P. Hsiue, Reza Farshchi, Mauricio Silva, Erik Zeegen, Rachel Thompson, Alexandra Stavrakis

**Affiliations:** a Department of Orthopaedic Surgery, UCLA, Santa Monica, CA, USA; b Department of Surgery, Albert Einstein Medical Center, Philadelphia, PA, USA; c UC San Diego School of Medicine, UCSD, La Jolla, CA, USA

**Keywords:** Total hip arthroplasty, Pediatric, Bearing surface trends

## Abstract

**Background:**

Long-term implant durability is a key concern when considering total hip arthroplasty (THA) in young patients. The ideal bearing surface used in these patients remains unknown. The purpose of this study was to analyze trends in THA bearing surface use from 2006 to 2016 using a large, pediatric national database.

**Methods:**

This was a retrospective review from January 1, 2006, to December 31, 2016, using the Kids’ Inpatient Database. International Classification of Diseases, 9^th^ revision and 10^th^ revision codes were used to identify patients who underwent THA and create cohorts based on bearing surfaces: metal-on-metal, metal-on-polyethylene, ceramic-on-polyethylene (CoP), and ceramic-on-ceramic (CoC). Annual utilization of each bearing surface and associated patient and hospital demographics were analyzed.

**Results:**

A total of 1004 THAs were identified during the 11-year study period. The annual number of THAs performed increased by 169% from 2006 to 2016. The mean patient age was 17.1 years. The most prevalent bearing surface used in 2006 was CoC (37.3%), metal-on-metal (31.8%) in 2009, and CoP in 2012 and 2016 (50.6% and 64.8%, respectively). From 2006 to 2016, utilization of CoP increased from 5.0% to 64.8%, representing a 1196% increase over the study period.

**Conclusions:**

The number of THAs performed in pediatric patients is increasing significantly. Although CoC was previously the most commonly used bearing surface in this patient population, CoP is currently the most common. Further investigation is needed to determine whether bearing longevity and clinical outcomes with CoP are superior to other bearing surfaces.

## Introduction

Over the past few decades, total hip arthroplasty (THA) has become one of the most commonly performed orthopedic procedures in the United States [1]. As THA is performed more frequently in young, active patients, concerns about wear-related premature failure become ever more significant. Traditionally, the consensus was that younger patients would benefit from hard-on-hard surface bearings—metal-on-metal (MoM) and ceramic-on-ceramic (CoC)—as these would improve durability and avoid the complications associated with polyethylene wear and associated osteolysis. However, the use of MoM bearings has fallen precipitously over the last decade because of early failures associated with metal wear debris aseptic lymphocyte-dominant vasculitis-associated lesions [2]. CoC bearing couplings remain a viable option; however, they are associated with noise and, rarely, with implant fracture, which can be a devastating complication [3]. At the same time, the wear characteristics of hard-on-soft bearing couplings such as ceramic-on-polyethylene (CoP) and metal-on-polyethylene (MoP) have improved significantly with the advent and widespread use of highly cross-linked polyethylene [4].

Considerations relating to implant longevity are especially important in the extremely young (<21 years) patient population. The most common indications for THA in pediatric patients and young adults include undertreated developmental dysplasia of the hip, femoral acetabular impingement, Legg-Calve-Perthes disease, slipped capital femoral epiphysis, avascular necrosis (AVN), inflammatory arthritis, and posttraumatic arthritis [5]. While several reconstructive procedures exist (including femoral and pelvic osteotomies for dysplasia and femoral head decompression for AVN), increasing evidence of the long-term durability of hip arthroplasty in adult populations has likely contributed to the rise of THA in pediatric patients in whom reconstructive procedures are inadequate—including femoral head collapse with AVN and Stulberg IV/V hips in Legg-Calve-Perthes [5]. But even as the number of THAs performed in the extremely young patient population increases, it is not known which bearing couplings are most frequently used and result in superior outcomes in this patient population.

The purpose of this study is to perform an epidemiological analysis of bearing surface utilization in THA in the extremely young (<21 years) patient population using a national administrative database. We hypothesize that over the course of the study period, there will be a decrease in the use of MoM bearings along with an increase in CoC and CoP.

## Material and methods

The study cohort was identified using the Kids’ Inpatient Database (KID) over an 11-year period (2006-2016), with data available for the years 2006, 2009, 2012, and 2016. The KID is a nationally representative database developed from all hospitals in the United States participating in the Healthcare Cost and Utilization Project (HCUP) and validated through a federal-state-industry partnership sponsored by the Agency for Healthcare Research and Quality (AHRQ). The KID contains inpatient data for patients younger than 21 years from 46 states plus the District of Columbia, covering approximately 80% of all pediatric discharges and 10% of uncomplicated hospital births from US hospitals, excluding rehabilitation and long-term acute care hospitals. The large sample size from the KID allows analysis of rare conditions. A stratified algorithm based on discharge weights reported by statewide HCUP contributors was designed to allow an estimation of nationally representative statistics. Available variables include demographic data, diagnoses, procedures, hospital characteristics, length of stay, and cost. As the KID is sufficiently deidentified of any personal health information or identifiers, this study was deemed exempt by the institutional review board at our institution.

Patients younger than 21 years who were admitted for THA were included in the study cohort. Patients were selected using the International Classification of Diseases, 9^th^ Revision (ICD-9) and 10^th^ Revision (ICD-10), procedure codes for THA. Patients were divided based on the type of bearing surfaces used for THA, identified using ICD-9 and ICD-10 codes for MoM, CoC, CoP, and MoP (Appendix Table 1). Annual incidence of each bearing surface type was calculated and used to generate trend lines for the study period. Individual hospitalization cost was calculated using diagnosis-related group codes multiplied by hospital-specific cost-to-charge ratios provided by the AHRQ. HCUP indices of the diagnosis-related group were then used to account for differences in severity of illness. Costs were then standardized for inflation using the yearly gross domestic product. Patients with missing data for any of the primary outcomes of interest were excluded.

All result sample sizes represented national annual estimates derived from individual discharge-level weights from the KID's random sampling design, using Stata’s survey data commands. Descriptive analysis was used to describe both baseline characteristics and outcome parameters within each comparison group. Categorical variables were presented as adjusted odds ratios and compared using the Chi-square statistic, except when there were less than 10 individuals, in which case the Fisher exact test was used. Continuous variables were reported using mean, 95% confidence interval, and *P* value. Analysis was completed using analysis of variance two-tailed Student’s t-test after ensuring normal distributions. For skewed distributions, continuous variables are presented as median (interquartile range) and analyzed using the Wilcoxon rank-sum test. Chi-square goodness of fit test was performed within each variable category. All statistical analyses were carried out comparing pediatric THA patients for each outcome of interest. Data were analyzed using Stata 15.0 (StataCorp LLC, College Station, TX). All tests were unpaired, and significance level was defined at *P* < .05.

## Results

**Figure 1:**
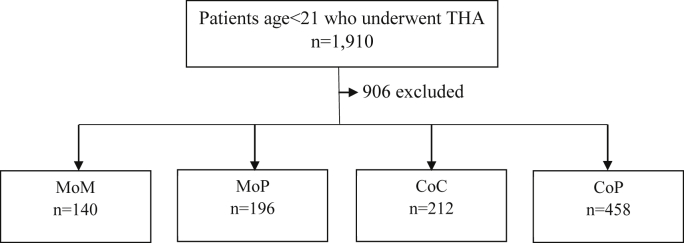
Flowchart of patient study population. Patients aged <21 years who underwent THA were separated into groups based on bearing types. Patients without bearing type designation were excluded.

**Figure 2:**
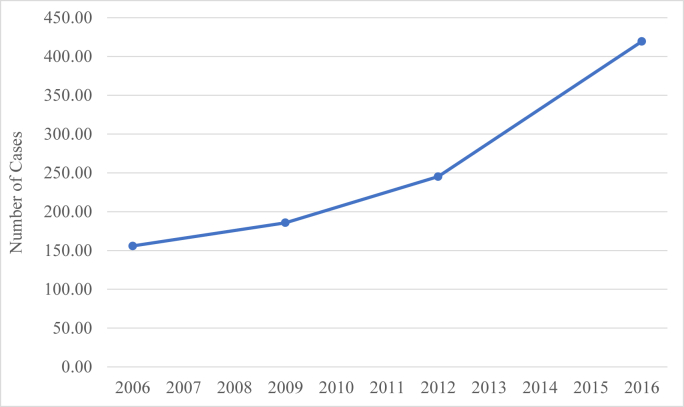
Incidence of pediatric total hip arthroplasty 2006-2016. There was an overall 169% increase in the number of pediatric THA cases. The increase was statistically significant if *P* < .05 using the Cochran-Armitage trend test.

A total of 1910 THAs were identified during the study period (Fig. 1). Bearing type used was available for 1004 (52.6%) of these THAs and were included for analysis. The annual number of THAs performed increased by 169%, from 156 in 2006 to 420 in 2016, which represented a statistically significant increase (*P* < .05) (Fig. 2). The mean patient age at implantation across the combined cohort was 17.2 years and did not significantly change over the study period (*P* = .4). The mean comorbidity score measured by the Elixhauser Comorbidity Index was 0.87, and there was no significant difference based on bearing type (*P* = .21). Average cost of hospitalization was $22,080.93. Mean index hospitalization costs were highest for MoP at $22,747.71 and lowest for CoP at $19,682.64 (*P* = .0082). From 2006 to 2016, length of stay decreased from 4.85 days to 3.19 days (*P* = .0007); there was no significant difference between age and Elixhauser Comorbidity Index or cost across the study period.

**Figure 3:**
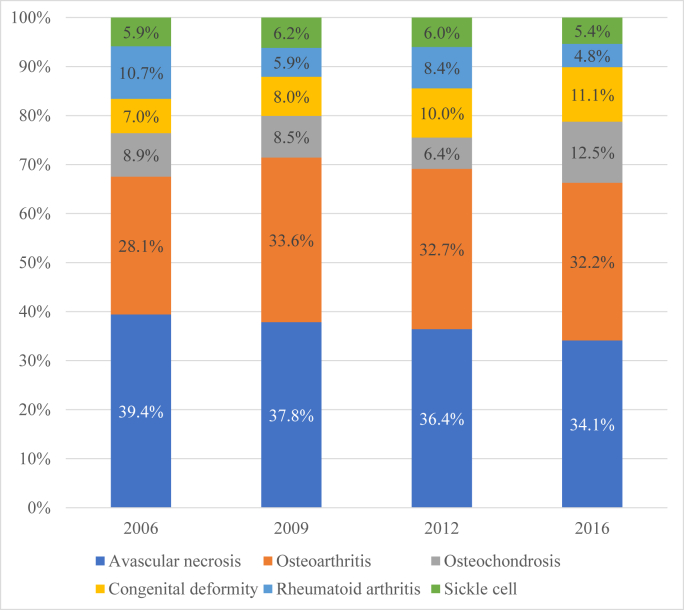
Indications over time for pediatric total hip arthroplasty: Avascular necrosis and osteoarthritis were the primary 2 causes for pediatric total hip arthroplasty. There was a decreased rate of avascular necrosis as reason for surgery over the study period.

Patient demographics are included in Table 1. THA were most likely to be performed at urban teaching hospitals (82%) and in the Southern region (38.5%) of the United States (Table 1). The most common indications for THA included AVN (36.6%), osteoarthritis (31.9%), and congenital hip deformity (9.3%). Figure 3 is a graphical representation of the breakdown of THAs performed each year of the study period by indication.

**Table 1:** Patient demographic characteristics.

Characteristics	MoM (%)	MoP (%)	CoC (%)	CoP (%)	*P* value
Sex					
Male	95 (67.6)	93 (48.3)	105 (49.3)	210 (46.1)	-
Female	45 (32.4)	99 (51.7)	108 (50.7)	246 (53.9)	.0059
Race					
White	62 (43.9)	96 (49.2)	104 (49.2)	234 (51.0)	.7228
Black	25 (18.0)	28 (14.6)	48 (22.5)	81 (17.6)	.4049
Hispanic	16 (11.1)	18 (9.1)	16 (7.4)	55 (12.1)	.4873
Asian	2 (1.1)	4 (2.1)	3 (7.4)	15 (3.3)	.4367
Other	7 (5.0)	12 (6.3)	8 (3.6)	27 (5.9)	.7673
Insurance					
Medicare	0 (0)	4 (2.2)	5 (2.4)	11 (3.1)	.5447
Medicaid	38 (26.9)	61 (31.2)	75 (35.5)	148 (32.2)	.6122
Private	94 (66.7)	109 (55.6)	110 (51.6)	270 (59.0)	.1669
Self	3 (2.4)	9 (4.5)	4 (2.0)	8 (1.8)	.5778
Hospital type					
Rural	4 (3.0)	4 (2.1)	2 (0.7)	3 (0.7)	.2576
Urban nonteaching	21 (15.3)	23 (11.5)	39 (18.4)	59 (12.9)	.392
Urban teaching	112 (79.6)	166 (84.7)	172 (80.8)	393 (85.8)	.4331
Hospital region					
Northeast	24 (17.4)	48 (24.8)	27 (12.9)	78 (17.0)	.1505
Midwest	33 (23.7)	55 (28.4)	44 (20.9)	97 (21.3)	.5092
South	52 (37.4)	46 (23.7)	97 (45.5)	176 (38.4)	.0159
West	30 (21.4)	45 (23.2)	44 (20.8)	107 (23.3)	.9479
Hospital size					
Small bed	29 (20.7)	24 (12.5)	33 (15.8)	81 (17.6)	.4615
Medium bed	20 (14.6)	46 (23.8)	45 (21.0)	69 (15.1)	.1724
Large bed	88 (62.6)	121 (62.1)	134 (63.2)	305 (66.7)	.8147
Income					
0-25th Percentile	52 (37.4)	45 (23.1)	60 (28.5)	124 (27.1)	.1198
25-50th Percentile	31 (21.9)	39 (19.9)	50 (23.7)	96 (20.9)	.8734
50-75th Percentile	26 (18.8)	51 (26.3)	51 (22.9)	101 (27.5)	.6084
75-100th Percentile	29 (20.9)	51 (26.3)	49 (22.9)	126 (27.5)	.5364

**Figure 4:**
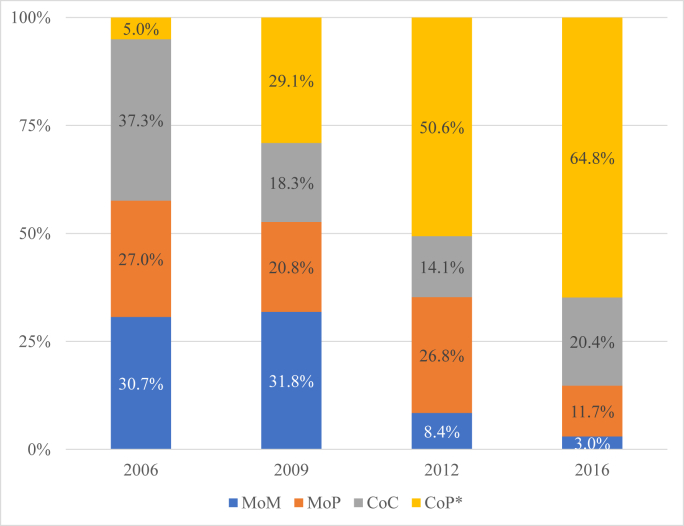
Ratio of bearing type in pediatric total hip arthroplasty from 2006 to 2016. There was a 57% decrease in rate of MoP used as well as a 90% decrease in the rate of MoM used. There was a 1196% increase in the rate of CoP used. ∗Statistically significant if *P* < .05 using Cochran-Armitage trend test.

Of the 1004 patients included for analysis, 140 (14.0%) were MoM, 196 (19.6%) MoP, 212 (21.1%) CoC, and 458 (45.6%) CoP (Fig. 1). From 2006 to 2016, utilization of CoP increased from 8 (5.0%) to 272 (64.8%), respectively, which represents a 1196% increase (Fig. 4). The most prevalent bearing surface in 2006 was CoC (37.3%), MoM (31.8%) in 2009, and CoP in 2012 and 2016 (50.6% and 64.8%, respectively).

## Discussion

To our knowledge, this is the first study to use a pediatric national administrative database to report on the indications for and bearing surface material utilization in THA performed in patients aged <21 years in the United States. We found a nearly 50% increase in THA nationally with a 1196% increase in CoP bearing surfaces between 2006 and 2016. Previously published results by Rajaee et al. on the trends of THA bearing surfaces in patients aged ≤30 years between 2009 and 2012 found that hard-on-hard bearing usage decreased from 2009 to 2012, whereas hard-on-soft bearing surface usage (MoP and CoP) increased [6]. However, the mean age in this cohort was 24 years, likely representing a different patient population.

The ideal bearing surface for THA in very young patients remains an important but unanswered question. Historically, it was felt that hard-on-hard bearings (ie, MoM and CoC) were advantageous for their increased longevity [7], but these surfaces have fallen out of favor in the ensuing years, as is made clear with this report.

The use of MoM bearings has declined sharply in all patient populations because of concerns associated with metal ion release leading to adverse reactions to metal debris [2,8,9]. MoP bearings with associated trunnionosis have also been linked with a higher risk for similar adverse reactions and reduced implant survival [10,11]. The decrease in the use of CoC bearings may have resulted from concerns over noise and the small but catastrophic risk of implant fracture [12,13]. The reported incidence of audible squeaking varies depending on head size and the brand of components studied; one study of large-diameter heads in CoC bearings found an increase in the incidence of squeaking after THA from 7.3% at 2 years to 17.4% at 5 years [14]. Examination of the Norwegian Arthroplasty Register between 1997 and 2017 including 31,479 CoP and 5790 CoC articulations found that the survivorship free from revision for ceramic fracture for CoC bearings was 99.8% at 10 years, but the hazard ratio for ceramic head fracture was 3.6 (95% confidence interval, 1.7 to 7.6) for CoC compared with CoP [15]. These results may have tempered interest in the use of CoC in the pediatric population. In addition, the improved wear characteristics and standardization of highly cross-linked polyethylene have further improved hard-on-soft articulations and may be contributing to the changes in national trends reported here [16,17].

This study has several limitations. The bearing surface used was not available for all THAs in the KID as this information is not required for compensation, which raises the possibility of selection bias. In addition, the database did not provide granular information on bearing types such head size, which generation of components were used, or the type of polyethylene used which limits our ability to draw bearing-specific conclusions. Clinical information was derived from a national administrative database populated with ICD-9 and ICD-10 codes, which are entered by hand and therefore subject to human error. However, physician and hospital compensation is based on these codes and therefore is generally highly accurate. Despite the general limitations associated with the use of a national administrative registry, the KID is validated through a federal-state-industry partnership sponsored by the AHRQ and is widely recognized as a reliable source of clinical information that has produced many peer-reviewed publications across a variety of specialties [[18], [19], [20]].

## Conclusions

The data presented strongly suggest that the incidence of THAs performed in patients aged <21 years is increasing and that CoP is increasingly popular among arthroplasty surgeons for this age group. Further investigation is warranted to determine if bearing longevity and clinical outcomes with CoP are superior to previously popular bearing surfaces in this particular patient population.

## Conflicts of interest

The authors declare the following financial interests/personal relationships which may be considered as potential competing interests: M. Silva has stocks or stock options in Imagen Technologiesand is in the medical/orthopaedic publications editorial/governing board of Journal of Haemophilia and Journal of Children Orthopaedics. E. Zeegen is a paid consultant for Zimmer Biomet and Smith & Nephew; has stock or stock options in Radlink, Inc.; received research support from Zimmer Biomet; is in the medical/orthopaedic publications editorial/governing board of Journal of Arthroplasty and Arthroplasty Today; and is a member of the Evidence Based Medicine Committee of American Association of Hip and Knee Surgeons. R. Thompson is a committee member of Pediatric Orthopaedic Society of North America and American Academy of Cerebral Palsy and Developmental Medicine.

## Figures and Tables

**Appendix Table 1:** Identifying patients of interest with International Classification of Diseases, 9th Revision and 10th Revision.

Procedures	ICD-9	ICD-10
THA	81.51	-a
MoM	00.75	0SR9019, 0SR901A, 0SR901Z, 0SRB019, 0SRB01A, 0SRB01Z
MoP	00.74	0SR9029, 0SR902A, 0SR902Z, 0SRB029, 0SRB02A, 0SRB02Z
CoC	00.76	0SR9039, 0SR903A, 0SR903Z, 0SRB039, 0SRB03A, 0SRB03Z
COP	00.77	0SR9049, 0SR904A, 0SR904Z, 0SRB049, 0SRB04A, 0SRB04Z
Reason for surgery		
Avascular necrosis	733.40, 733,42, 733.49	M87.9, M87.00, M87.08, M87.09, M87.10, M87.19, M87.20, M87.28, M87.29, M87.30, M87.38, M87.39, M87.80, M87.88, M87.89, T38.0X5, M87.188, M87.050, M87.051, M87.052, M87.059, M87.150, M87.151, M87.152, M87.159, M87.250, M87.251, M87.252, M87.256, M87.350, M87.351, M87.352, M87.353, M87.850, M87.851, M87.852, M87.859
Osteoarthritis	715.15, 715.25, 715.35, 715.95, 715.10, 715.20, 715.30, 715.00, 715.80, 715.90	M1255, M16.0, M16.10, M16.11, M16.12, M16.4, M16.50, M16.51, M16.52, M16.6, M16.7, M16.9
Rheumatoid arthritis	714.30, 714.31, 714.32, 714.33, 714.0	M0505, M0515, M0525, M0535, M0545, M0555, M0565, M0575, M0585, M0765, M0805, M0825, M0845, M0885, M0895, M1205, M1215, M1225, M1235, M1245, M1285, M1315, M1385, M1465, M1485, M0500, M0510, M0520, M0530, M0540, M0550, M0560, M0570, M0580, M0760, M0800, M0820, M0840, M0880, M0890, M1200, M1210, M1220, M1230, M1240, M1280, M1310, M1380, M1460, M1480, M0509, M0519, M0529, M0539, M0549, M0559, M0569, M0579, M0589, M0769, M0809, M0829, M0849, M0889, M0899, M1209, M1219, M1229, M1239, M1249, M1289, M1319, M1389, M1469, M1489, M05.9, M08.3, M12.9, M13.0
Congenital deformity	755.63, 755.69	Q65.00, Q65.01, Q65.02, Q65.1, Q65.2, Q65.30, Q65.31, Q65.32, Q65.4, Q65.5, Q65.6, Q65.81, Q65.82, Q65.89, Q65.9, M16.2, M16.30, M16.31, M16.32
Osteochondrosis	732.1, 732.2, 732.6, 732.7, 732.9	M91.0, M91.10, M91.11, M91.12, M91.20, M91.21, M91.22, M91.30, M91.31, M91.32, M91.40, M91.41, M91.42, M91.80, M91.81, M91.82, M91.90, M91.91, M91.92, M93.001, M93.002, M93.003, M93.011, M93.012, M93.013, M93.021, M93.022, M93.023, M93.031, M93.032, M93.033, M93.80, M93.811, M93.812, M93.819, M93.821, M93.822, M93.829, M93.831, M93.832, M93.839, M93.841, M93.842, M93.849, M93.851, M93.852, M93.859, M93.861, M93.862, M93.869, M93.871, M93.872, M93.879, M93.88, M93.89, M94.1, M94.9, M93.20, M93.251, M93.252, M93.259, M93.28, M93.29, M93.90, M94.251, M94.252, M94.259, M94.351, M94.352, M94.359
Sickle cell	282.60, 282.61, 282.62, 282.63, 282.64, 282.68, 282.69	D57.00, D57.01, D57.02, D57.03, D57.09, D57.1, D57.20, D57.211, D57.212, D57.213, D57.218, D57.219, D57.3, D57.40, D57.411, D57.412, D57.413, D57.418, D57.419, D57.42, D57.431, D57.432, D57.433, D57.438, D57.439, D57.44, D57.451, D57.452, D57.453, D57.458, D57.459, D57.80, D57.811, D57.812, D57.813, D57.818, D57.819

ICD-9, International Classification of Diseases, 9th Revision; ICD-10, International Classification of Diseases, 10th Revision.

a ICD-10 schema integrates THA and bearing type with each procedure.

## Appendix A Supplementary data

Conflict of Interest Statement for Thompson

Conflict of Interest Statement for Farshchi

Conflict of Interest Statement for Chen

Conflict of Interest Statement for Hart

Conflict of Interest Statement for Stavrakis

Conflict of Interest Statement for Hsiue

Conflict of Interest Statement for Silva

Conflict of Interest Statement for Zeegen
