# Supplementary material for: National Trends in Total Hip Arthroplasty Bearing Surface Usage in Extremely Young Patients Between 2006 and 2016
Source: Arthroplast Today. 2021 Jul 9;10:51–6. doi: 10.1016/j.artd.2021.05.017 (PMC8283017; doi:10.1016/j.artd.2021.05.017)
Supplement: Conflict of Interest Statement for Silva [file mmc7.pdf]

CONFLICT OF INTEREST STATEMENT

American Association of Hip and Knee Surgeons

(Adopted from the American Academy of Orthopaedic Surgeons disclosure statement)

The following form **must be filled out completely and submitted by each author (example, 6 authors, 6 forms). All items require a response. If there is no relevant disclosure for a given item, enter "*None.*"**

National Trends in Total Hip Arthroplasty Bearing Surface Usage in Extremely Young Patients Between 2006 and 2016

Manuscript Title

1. Royalties from a company or supplier: None

2. Speakers bureau/paid presentations for a company or supplier: None

3A. Paid employee for a company or supplier: None

3B. Paid consultant for a company or supplier: None

3C. Unpaid consultants for a company or supplier: None

4. Stock or stock options in a company or supplier: Imagen Technologies

5. Research support from a company or supplier as a Principal Investigator: None

6. Other financial or material support from a company or supplier: None

7. Royalties, financial or material support from publishers: None

8. Medical/Orthopaedic publications editorial/governing board: Journal of Haemophilia, Editorial Board. Journal of Children Orthopaedics, Editorial Board.

9. Board member/committee appointments for a society: None

Each author must sign AND print or type his/her name, date and submit a separate form

In addition, one BLINDED Conflict of Interest form (no author names used) should be submitted per manuscript with all author disclosures.

Mauricio Silva, MD		2/10/2021
Author Name (Print or Type)	Author Signature	Date
